# Extracellular vesicle miR-32 derived from macrophage promotes arterial calcification in mice with type 2 diabetes via inhibiting VSMC autophagy

**DOI:** 10.1186/s12967-022-03502-8

**Published:** 2022-07-06

**Authors:** Jingsong Cao, Cong Chen, Qian Chen, Yan Gao, Zhibo Zhao, Qing Yuan, Anqi Li, Shiqi Yang, Yuqi He, Xuyu Zu, Jianghua Liu

**Affiliations:** 1 The First Affiliated Hospital, Institute of Clinical Medicine, Department of Endocrinology and Metabolism, Hengyang Medical School, University of South China, Hengyang, 421000 Hunan China; 2 The First Affiliated Hospital, Department of Laboratory Medicine, Hengyang Medical School, University of South China, Hengyang, 421000 China; 3 The First Affiliated Hospital, Institute of Clinical Medicine, Department of Tumor Research, Hengyang Medical School, University of South China, Hengyang, 421000 China

**Keywords:** Macrophage, EVs, miR-32, Diabetes, Vascular calcification

## Abstract

**Background:**

The development of diabetes vascular calcification (VC) is tightly associated with the inhibition of vascular smooth muscle cell (VSMC) autophagy. Previously, our team found that miR-32-5p (miR-32) promotes macrophage activation, and miR-32 is expressed at higher level in the plasma of patients with coronary calcification. However, whether miR-32 mediates the function of macrophages in type 2 diabetes (T2D) VC is still unclear.

**Methods:**

Wild-type (WT) and miR-32^−/−^ mice were used in this study. qRT-PCR and western blotting were used to analyze gene expression. Flow cytometry was used to analyze the influence of glucose concentration on macrophage polarization. Nanoparticle tracking analysis (NTA), transmission electron microscopy, and confocal microscopy were used to identify macrophage extracellular vehicles (EVs). Immunofluorescence, in situ hybridization (ISH), immunohistochemistry, and alizarin red staining were used to analyze the influence of macrophage EVs on autophagy and calcification of the aorta of miR-32^−/−^ mice. A luciferase assay was used to analyze the effect of miR-32 on myocyte enhancer factor 2D (Mef2d) expression. Co-IP combined with mass spectrometry (MS) and transcriptome sequencing was used to analyze the signalling pathway by which Mef2d acts in VSMC autophagy.

**Results:**

We found that high glucose conditions upregulate miR-32 expression in macrophages and their EVs. Importantly, macrophages and their EVs promote VSMC osteogenic differentiation and upregulate miR-32 expression in VSMCs. Moreover, miR-32 mimics transfection promoted osteogenic differentiation and inhibited autophagy in VSMCs. In vitro and in vivo experiments showed that Mef2d is the key target gene of miR-32 that inhibits VSMC autophagy. Furthermore, MS and transcriptome sequencing found that cGMP-PKG is an important signalling pathway by which Mef2d regulates VSMC autophagy. In addition, after T2D miR-32^−/−^ mice were injected with macrophage EVs via the caudal vein, miR-32 was detected in aortic VSMCs of miR-32^−/−^ mice. Moreover, autophagy was significantly inhibited, and calcification was significantly enhanced in aorta cells.

**Conclusions:**

These results reveal that EVs are the key pathway by which macrophages promote T2D VC, and that EVs miR-32 is a key cause of autophagy inhibition in VSMCs.

**Supplementary Information:**

The online version contains supplementary material available at 10.1186/s12967-022-03502-8.

## Introduction

Diabetes easily induces microvascular and macrovascular complications that lead to peripheral vascular disease [1]. Vascular calcification (VC) is an important clinicopathological feature of diabetes and is considered as a major independent risk factor for cardiovascular diseases [2]. In the process of VC, at least 4 kinds of cells may lead to VC. These cell types include pericytes in microvessels, pericyte-like calcifying vascular cells in the aortic intima, smooth muscle cells (SMCs) in the media, and myofibroblasts in the adventitia [3]. Among the 4 cell types, local smooth muscle cells are an important source of calcifying vascular cells [3–5]. The key to VSMC calcification is the transformation from a contractile phenotype to an osteoblast-like phenotype [6]. However, the mechanism of VSMC calcification is still unclear.

Our previous research determined that the expression of miR-32 is increased in VSMCs during the progression of vascular calcification, and miR-32 was expressed at high level in the plasma of VC patients compared with non-VC patients [7]. Interestingly, we further found that miR-32 is also involved in the activation of microglia, which are resident macrophages in the central nervous system [8]. Therefore, exploring the relationship and mechanisms among macrophages, miR-32 and VSMC calcification is meaningful.

Macrophages are the major participators of innate immunity and exist in all human tissues, including the aortic wall [9]. In response to infection, peripheral monocytes, derived from the bone marrow, are the major source of macrophages in the aortic wall [10]. In the plaques of human and mouse models, macrophages are abundant immune cells, and they are the primary cell type among total plaque cells [11, 12]. Infiltrated macrophages polarize to the proinflammatory M1 phenotype and secrete inflammatory factors and exosomes, leading to increased plaque stability [13, 14]. Macrophage polarization is a response to environmental stimulation and involves coordinated metabolic and transcriptional rewiring [15]. In diabetes, high glucose induces M1 macrophage polarization and the secretion of exosomes to regulate the development of cardiovascular diseases [16, 17]. Recent research found that the exosomes recovered by centrifugation include oncosomes, ectosomes, microvesicles and membrane vesicles, so the production is better called EVs [18]. However, the roles of macrophages in T2D VC and how miR-32 increases in VSMCs are still unclear.

Autophagy is a catabolic process in cells that occurs via the lysosomal degradation pathway [9]. In the autophagy pathway, phagophore formation is a vital step in the formation of autophagosomes [19]. This process is regulated by several autophagy-related (Atg) proteins, including Beclin1 (Becn-1), Atg6 and Atg38 [20]. Following the development of autophagosomes, Atg proteins (e.g., Atg5, Atg16l1 and Atg7) also regulate their expansion and maturation of autophagosomes [21], while other proteins (e.g., sequestosome 1 (p62)) mediate autophagosome engulfment by lysosomes [22]. Diabetes mellitus is a chronic heterogeneous metabolic disorder [23]. Impaired autophagy mediates VSMC phenotypic conversion [24] and further accelerates VSMC calcification [25]. Clinically, insufficient autophagy activation is considered as one phenotypic feature of calcific aortic valve stenosis [26]. These results suggest that autophagy is closely associated with macrophage polarization and VC.

Therefore, this study aims to explore the mechanism by which macrophages promote VC in T2D. To address this aim, we found that miR-32 promotes M1 macrophage polarization under high glucose conditions and demonstrated that the secretion of EVs miR-32 is the major pathway by which macrophages promote VC in T2D through Mef2d/cGMP-PKG-mediated VSMC autophagy. Then, we verified the results in a T2D miR-32^−/−^ mouse model. These findings illustrate an important mechanism by which macrophages promote VC in T2D and identify a candidate biomarker of T2D VC.

## Materials and methods

### Cell lines culture

RAW264.7 and 293 T cells were purchased from the National Infrastructure of Cell Line Resource (China Center for Type Culture Collection) and were cultured in high- or low-glucose Dulbecco’s modified Eagle’s medium (DMEM, Gibco BRL, Grand Island, USA) with 10% fetal calf serum (FBS, Gibco, Australia) and 100 U/ml penicillin–streptomycin at 37 ℃ and 5% CO_2_.

### Mouse VSMCs separation and culture

Aortas were obtained from 8 to 10-week-old mice. Extravascular fat was removed from the aortas, and the aortas were cut open along the vessel lumen. Then, the tunica adventitia and endothelium of the vascular wall were removed. After cutting the aortas into small pieces, 1 ml culture medium (DMEM with 15% FBS and 1% penicillin–streptomycin) was added, and the sample was centrifuged at 250 g for 1 min. The precipitate was resuspended in 2 ml culture medium and transplanted into a T25 bottle (2 mice/bottle). After culturing at 37 ℃ and 5% CO_2_ overnight, 2 ml culture medium was added, and the culture media was changed every 2 days. Finally, VSMCs were collected for further culture or analysis.

### Macrophage EVs assay

EVs extraction from cell culture media was performed using a Total Exosome Isolation Reagent Kit (Thermo Fisher Scientific Inc., Waltham, MA USA) with some modifications. The dish with 70–80% confluent RAW264.7 cells was washed with 1 × PBS 3 times, serum-free medium was added, and the cells were cultured at 37 ℃ and 5% CO_2_ for 24 h. The medium was subsequently collected and centrifuged at 2000 g for 30 min followed by 10,000 g for 30 min. Then, the supernatant was collected and incubated with a half volume of EVs isolation reagent at 4 °C overnight. Finally, the mixture was centrifuged at 10,000 g for 1 h, and the precipitate was resuspended in PBS. EVs were detected using nanoparticle tracking analysis (NTA), transmission electron microscopy and western blotting or were stored at − 80 ℃.

For the in vitro experiment, VSMCs were treated with EVs at a ratio of 1:1,000 and cultured at 37 ℃ and 5.0% CO_2_ for 48 h. For the in vivo experiment, T2D mice were injected with 1.0 × 10^9^ EVs via the tail vein once per week for two weeks.

### Generation of T2D mice

8- to 10-week-old mice (C57/BL6 background) were fed a chow fat diet (CFD) or a high-fat diet (HFD) with 10% (TP23102) or 45% (TP 23100) of the energy from fat, respectively (Trophic Animal Feed High-tech Co., Ltd., Jiangsu, China). Five weeks later, an intraperitoneal insulin tolerance test (IPITT) assay was performed to detect changes in blood glucose after insulin (Sigma Chemical, USA) injection at 0, 15, 30, 60 and 120 min. Then, streptozotocin (STZ, Sigma Chemical, USA) was intraperitoneally injected at 25 mg/kg for 3 consecutive days. Blood glucose was measured from tail bleeds using a glucometer (Sannuo Biosensing Co., Ltd., Hunan, China) at specified time points every week, and HFD-fed mice with blood glucose ≥ 11.1 mM or significantly higher than CFD-fed mice were considered to have T2D. Serum insulin was detected using a Mouse INS ELISA Kit (Feiya Biotechnology Co., Ltd., China). The Homeostasis Model Assessment-Insulin Resistance (HOMA-IR) index was calculated according to the formula HOMA-IR = Fasting blood glucose (mg/dL) × fasting serum insulin (mU/mL)/405.

### Transwell assay

The influence of macrophages on VSMCs was assessed using Transwell plates (Corning Incorporated, NY, USA) containing a 0.4 μm pore membrane. RAW264.7 cells (1 × 10^5^) were plated into the upper chamber, and equal numbers of VSMCs were plated into the lower surface. After culturing at 37 ℃ and 5.0% CO_2_ for 48 h, VSMCs were collected for further analysis. Otherwise, the RAW 264.7 upper chamber was first treated with 1 μM 3,3’-dioctadecyloxcarbocyanine perchlorate (Dio) (MedChemExpress, NJ, USA) for 30 min or Dio for 30 min and 1 μM GW4869 (MedChemExpress) for 1 h, washed 3 times with culture medium and cocultured with VSMCs at 37 ℃ and 5.0% CO2 for 48 h.

### Transcriptome sequencing

VSMC was cocultured with or without RAW264.7 through 6-well transwell plates. After culturing at 37 ℃ and 5.0% CO_2_ for 48 h, VSMC was collected and then analyzed via transcriptome sequencing by RiboBio (Guangzhou, China). Simply, after assessed the quantity integrity of RNA yield with K5500 (Beijing Kaiao Technology Development Co., Ltd, Beijing, China) and the Agilent 2200 Tape Station (Agilent Technologies, CA, USA) seperately, the mRNA was treated according the requirements of Illumina. The purified library products were evaluated using the Agilent 2200 TapeStation and Qubit (Thermo Fisher Scientifi). The libraries were sequenced by Illumina (Illumina, CA, USA). Raw fastq sequences were treated with Trimmomatictools(v 0.36) using the following options: TRAILING:20, SLIDINGWINDOW:4:15 MINLEN:52, to remove trailing sequences below a phred quality score of 20 and to achieve uniform sequence lengths for downstream clustering processes. Sequencing read quality was inspected using the FastQC software. Adapter removal and read trimming were performed using Trimmomatic. Sequencing reads were trimmed from the end (base quality less than Q20) and filtered by length (less than 52). Paired-end reads were aligned to the mouse reference genome mm10/hg19/rn6 with HISAT2. HTSeq v0.12.4 was used to count the reads numbers mapped to each gene.

### MS analysis of MEF2D interaction protein

RAW264.7 was cultured in high-glucose condition. To remove nonspecific combination proteins, 1 ml of macrophage protein extract was incubated with 100 μl of Protein A/G Plus Agarose (Santa Cruz Biotechnology, Inc., CA, USA), which had been washed twice and resuspended at a 50% concentration in PBS. After detecting the protein concentration, 2 μg rabbit anti-mouse Mef2d antibody was added to 500 μg protein extract, gently mixed by hand and then incubated overnight at 4 ℃. On the second day, 50 μl agarose at a 50% concentration was added and incubated at 4 ℃ for 2 h, followed by concentration at 800 g for 5 s. Finally, the agarose was collected and then identified via MS by BGI. Simply, the sample protein was separated by SDS-PAGE, and followedin gel digestion. 1/3 of total peptides were separated and analyzed with a nano­UPLC (EASYnLC1200) coupled to a Q Exactive HFX Orbitrap instrument (Thermo Fisher Scientific) with a nano­electrospray ion source. Vendor’s raw MS files were processed using MaxQuant software (Version 1.6.15.0) on a linux OS server (debian­9).

The Antibodies information was showed in Table 2.

### Hematoxylin and eosin (HE) analysis

Five randomly selected mice per group were subjected to deep anesthesia using 10% chloral hydrate (3.5 ml/kg, I.P.) and the tissues were extracted and fixed in 4% paraformaldehyde. Following fixation in paraffin, the tissues were cut into consecutive 4 μm thick sections on a microtome, and the paraffin sections were transferred to poly-L-lysine-coated slides. The paraffin sections were processed in turn as baked at 60 ℃ for 12 h, incubated in xylene (Sinopharm, Shanghai, China) 3 times for 20 min, alcoholic (Sinopharm) gradient treated (100%, 95%, 85% and 75%) for 5 min per gradient, and soaked in double distilled water (ddH_2_O) for 5 min. Then, the sections were used for further detection as follows.

HE staining was performed for the histopathological examination. The sections were processed in turn for hematoxylin staining for 1 min using ddH_2_O washes, eosin staining for 30 s with ddH_2_O washes, alcoholic gradient dehydration (95–100%) for 5 min per gradient, and incubated in xylene 2 times for 10 min. The sections were then exposed to a neutral resin (Sinopharm) sealing sheet and observed using a DAB kit (ZSGB-BIO).

### Immunohistochemistry analysis

Deparaffinated sections of the aorta were prepared by heating the sample for 20 min, cooling the sample at room temperature, and then washing the sample 3 times with 0.01 M PBS (pH 7.2–7.6) for 3 min. Endogenous peroxidase activity was blocked by incubating the sections in 1.0% periodic acid for 10 min and then washing them 3 times with 0.01 M PBS for 3 min. The sections were incubated with rabbit anti-mouse iNOS at 4 ℃ overnight. After the sections were washed 3 times with PBS for 5 min, they were incubated with anti-rabbit IgG-HRP at 37 ℃ for 30 min. They were then washed 3 times with PBS for 5 min, and further treated with Metal Enhanced DAB Substrate Kit (Solarbio) at room temperature for 1–5 min. Following hematoxylin staining, alcoholic dehydration, xylene treatment and application of a neutral resin sealing sheet, the sections were visualized using a DAB kit (ZSGB-BIO). The antibodies information was showed in Table 2.

### In situ hybrization (ISH)

The experiment was carried out with mmu-miR-32-5p ISH Detection kit I (Boster, Wuhan, China). Simply, Deparaffinated sections of the aorta were prepared by heating the sample for 20 min, followed Inactivation of endogenous enzymes 3% H_2_O_2_ at room temperature for 10 min. Then, the sections incubated with 3% citric acid-diluted pepsin at 37 ℃ for 15 min, treated with 1% paraformaldehyde at room temperature for 5 min, pre-hybridized with prehybridization liquid at 42 ℃ for 4 h, hybridized with hybridization liquid at 42 ℃ for overnight, blocking at 37 ℃ for 30 min, incubated with biotinylated rats’ anti-digoxin at 37 ℃ for 1 h, treated with SABC at 37 ℃ for 20 min, incubated with biotinylated peroxidase at 37 ℃ for 20 min. Finally, the sections were visualized using a DAB kit (ZSGB-BIO) and stained with hematoxylin.

### Immunofluorescence analysis

Deparaffinated aortic sections were prepared as follows: infiltrated in EDTA buffer (pH 9.0) and heated for 24 min, cooled to room temperature and washed 3 times with 0.01 M PBS (pH 7.2–7.6) for 3 min, infiltrated in sodium borohydride for 30 min with ddH_2_O washes for 5 min, infiltrated in 75% alcohol for 30 s, stained Sudan black for 15 min with ddH_2_O washes for 5 min, blocked in 10% FBS for 60 min, incubated with mouse p62 monoclonal antibody or rabbit anti-mouse LC3 antibody at 4 ℃ for overnight with PBS washes 3 times for 5 min each, incubated with anti-rabbit IgG conjugated CoraLite488 or anti-rat IgG conjugated CoraLite594 at 37 ℃ for 1 h, washed 5 min with PBS for 3 times and DAPI stained at 37 ℃ for 10 min. After washing with PBS and applying the glycerin sealing sheet, the sections were visualized using an inverted fluorescence microscope (OLYMPUS IX71). The antibodies information was showed in Table 2.

### Identification of aorta calcification

Deparaffinated sections of the aorta were fixed in 95% alcohol for 15 min, and washed with 0.01 M PBS (pH 7.2–7.6) 3 times. The sections were stained with 50 μl Alizarin Red S solution (Solarbio) for 5 min and then washed with ddH_2_O for 3 times. After baking at 60 ℃ for 30 min and incubating in xylene 2 times for 10 min each, the sections were sealed with neutral resin (Sinopharm) sealing sheet and visualized using a DAB kit (ZSGB-BIO).

### Transfection

After the cells reached 70% confluence, transfection mixture (100 μl DMEM, 2 μl Lipofectamine 3000 (Thermo), 25 nmol miR-32 mimics or 25 nmol miR-32 inhibitor or 12 pmol si-Mef2d or 1 ug pDoubleEx-EGFP-Mef2d recombinant plasmid) was added to every 1 ml culture media, which was preincubated at room temperature for 20 min. The treated cells were cultured at 37 ℃ and 5% CO_2_ for 24 h or 48 h.

### Luciferase assay

The wild-type and mutant sequences of the 3′ untranslated region (UTR) of Mef2d were chemically synthesized, cloned into the pSicheck-2 luciferase reporter plasmid (BGI, China) and named Mef2d 3′ UTR WT (wild type) and Mef2d 3′ UTR Mut (mutant). Then, 500 ng Mef2d 3′ UTR WT or Mef2d 3′ UTR Mut and 25 nM miR-32 mimics or NC (RiboBio Co. Ltd.,China) were mixed with Lipofectamine 3000 and then transfected into RAW264.7 cells. The cells were cultured at 37 °C and 5% CO_2_ for 48 h. After the cells were harvested, a luciferase assay was performed using the Dual-Luciferase Reporter Assay System Kit (Promega, San Luis Obispo, CA, USA) at Turner BioSystems (Sunnyvale, CA, USA).

### Flow cytometry

Peripheral blood or RAW264.7 cells were divided into three groups (50 μl/sample): blank group, isotype control group and experimental. The isotype control group received Rat IgG2a Kappa-FITC, Rat IgG2b Kappa-PerCP-Cy5.5, and Rat IgG2a Kappa-APC. The experimental group received CD86 monoclonal antibody-FITC, CD11b monoclonal antibody-PerCP-Cy5.5 and F4/80 monoclonal antibody-APC. All groups were incubated at room temperature for 30 min. If the sample was peripheral blood, a treatment step was added for incubation with 1 ml red blood cell lysate (BD Biosciences) for 10 min. Then, the samples were resuspended in 1 ml PBS and concentrated at 250 g for 5 min. Finally, the pellet was resuspended in 300 μl PBS and detected using BD FACS AriaTM II. The antibodies information was showed in Table 2.

### RNA extraction and cDNA synthesis

Total RNA from cells or tissues was extracted using the RNA simple Total RNA Kit (TianGen Biotech (Beijing) Co., Ltd., China) or the miRNA Purification Kit (CWBIO, Beijing, China), Total cDNA was synthesized using the Revert Aid First Strand cDNA Synthesis Kit (Thermo), and miRNA was synthesized using the Mir-XTM miRNA First Strand Synthesis Kit (Takara Biomedical Technology (Beijing) Co., Ltd. Beijing, China).

### qRT-PCR

The 20 μl reaction volume of qRT-PCR contained 10 μl 2 × SYBR Green PCR Mastermix (Takara), 1 μl forward primer, and 1 μl reverse primer (Table 1), 1 μl cDNA template and 7 μl ddH_2_O. The reaction program was as follows: 95 °C for 2 min; 40 cycles at 95 °C for 15 s, and 60 °C for 30 s. The experiment was performed in a LightCycler 480 II. GAPDH is used as internal reference gene, and the results were calculated with the formula:2^−ΔΔCt^.

### Western blotting

Protein samples were separated by SDS-PAGE, and an 8% separation gel was prepared and electrophoresed at 80 V for 20 min, followed by 120 V for 90 min. Then, the protein was transferred onto a polyvinylidene fluoride (PVDF) membrane using a semi-dry transfer apparatus (Bio-Rad Laboratories, Inc., CA, USA) according to the manufacturer’s instructions. The membrane was then blocked at room temperature for 1 h in blocking buffer (TBS containing 5% nonfat powdered milk and 0.1% Tween-20), and then incubated with primary antibody overnight at 4 ℃. After the membrane was washed 3 times for 5 min each, it was incubated with secondary antibody linked horseradish peroxidase at room temperature for 40 min and washed 2 times for 20 min each. Finally, the membrane was developed using an Immobilon Western Chemiluminescent HRP Substrate kit (EMD Millipore Corporation, MA, USA) and analyzed using ChemiDoc ™ XRS + (Bio-Rad). The antibodies information was showed in Table 2.

### Bioinformatics analysis

Cytoscape 3.7.1 software was used to search for the interacting proteins of MEF2D. The DAVID database was used for gene-annotation enrichment analysis (https://david.ncifcrf.gov/summary.jsp). The Gene Ontology database was used to search for gene annotation (http://geneontology.org/). The miRDB database was used to search the for genes of miR-32 (https://www.mirbase.org/). Venn diagram analysis was used to identify the common genes (https://bioinfogp.cnb.csic.es/tools/venny/index.html).

### Statistical analysis

All experiments were repeated at least 3 times. The data was analyzed using GraphPad Prism 7.00 software, and the results are shown as the mean ± s.e.m. Student’s t test or one-way ANOVA with Bonferroni correction was used to assess statistical significance. Multiparameter analysis of clinical data was performed using logistic regression analysis. p < 0.05 or p < 0.01 were considered significant or very significant, respectively.

## Results

### Macrophage EVs promoted VSMC osteogenic differentiation under high glucose conditions

**Fig. 1:**
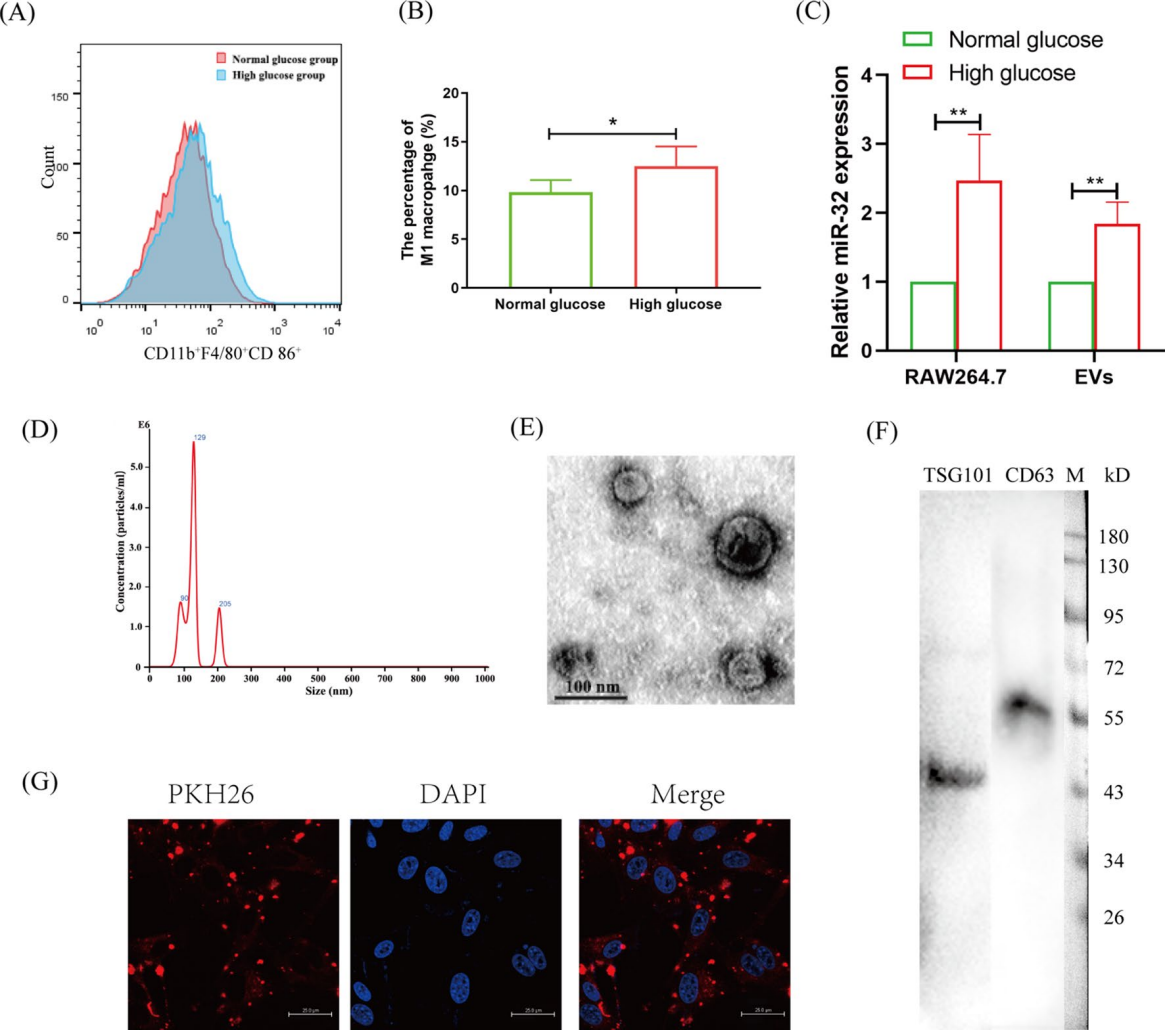
High-glucose condition promotes macrophage secreting miR-32 EVs. **A**, **B** Flow cytometry analyzed the influence of high-glucose for M1 macrophage polarization (**A**), and statistical analysis (**B**). **C** qRT-PCR analyzed the influence of high-glucose for miR-32 expression in macrophage and EVs. **D** NTA analyzed the quantity and diameter of EVs. **E** Western blotting identified the expression of EVs marker protein, TSG101 and CD63. **F** Transmission electron microscope analysis EVs morphology. **G** Confocal microscopy observed VSMC engulfed macrophage EVs, which were stained with red fluorescence. The experiment was repeated 3 times (*n* = 3), *p < 0.05 and **p < 0.01. Data was shown as means ± *s.e.m*

Under high glucose conditions, macrophages preferentially polarized to the M1 phenotype (Fig. 1A, B), and miR-32 expression was significantly higher than that under normal glucose conditions (Fig. 1C). Interestingly, we found that miR-32 expression was upregulated in macrophage EVs under high glucose condition compared with normal glucose conditions (Fig. 1C). To further illustrate whether macrophages regulate VSMC biofunction through the EVs pathway, EVs from macrophages were extracted and identified (Fig. 1D–G). Macrophage EVs had a diameter of 130.1 ± 36.2 nm (Fig. 1D) and a typical membrane structure (Fig. 1E). They expressed the marker genes of EVs TSG101 and CD63 (Fig. 1F). EVs can be taken up by VSMCs (Fig. 1H). These findings suggest that EVs may be an important pathway for the communication between macrophages and VSMCs.

**Fig. 2:**
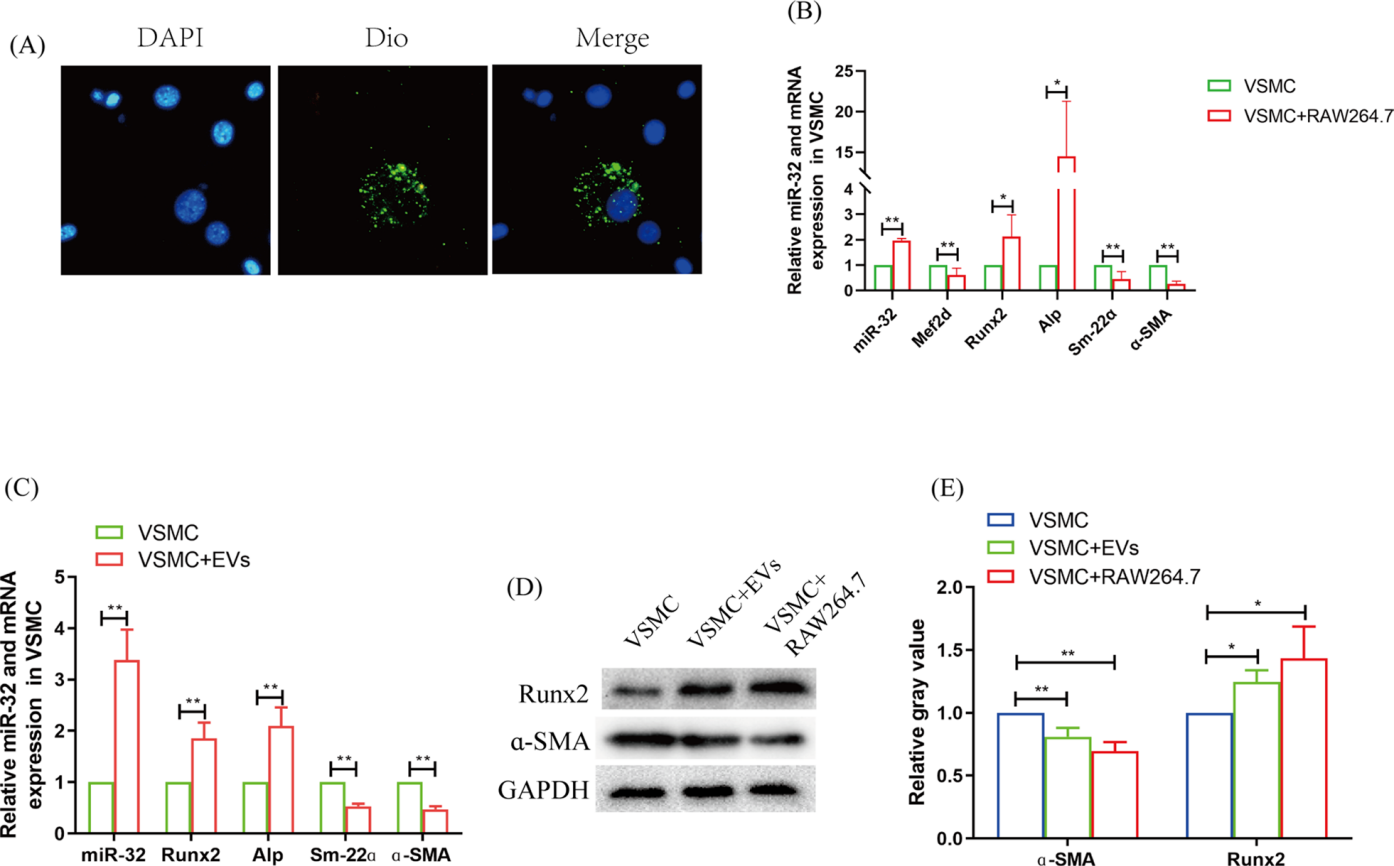
Macrophage EVs promotes VSMC osteogenic differentiation. **A** fluorescence microscope observes VSMC uptaking macrophage extracellular vesicles. **B** qRT-PCR analyzed the influence of macrophage for VSMC osteogenic differentiation. **C** qRT-PCR analyzed the influence of macrophage EVs for VSMC osteogenic differentiation. **D**, **E** Western blotting analyzed VSMC osteogenic differentiation after macrophage and EVs (**D**), and statistical analysis (**E**). The experiment was repeated 3 times (*n* = 3), *p < 0.05 and **p < 0.01. Data was shown as means ± *s.e.m*

Furthermore, VSMCs can take up macrophage extracellular vesicles in a transwell assay with a 0.4 μm pore membrane (Fig. 2A). After VSMCs were cocultured with RAW264.7, the marker genes of calcification, runt-related transcription factor 2 (Runx2) and alkaline phosphatase (Alp), increased significantly; the marker genes of SMC, smooth muscle 22-alpha (Sm-22ɑ) and alpha-smooth muscle actin (ɑ-SMA), decreased significantly (Fig. 2B, D, E). Interestingly, the results were replicated after VSMCs were treated with EVs secreted by macrophages in high glucose (Fig. 2C–E). These findings suggest that EVs promote VSMC osteogenic differentiation.

### miR-32 in macrophage EVs inhibited VSMC autophagy and promoted its osteogenic differentiation

**Fig. 3:**
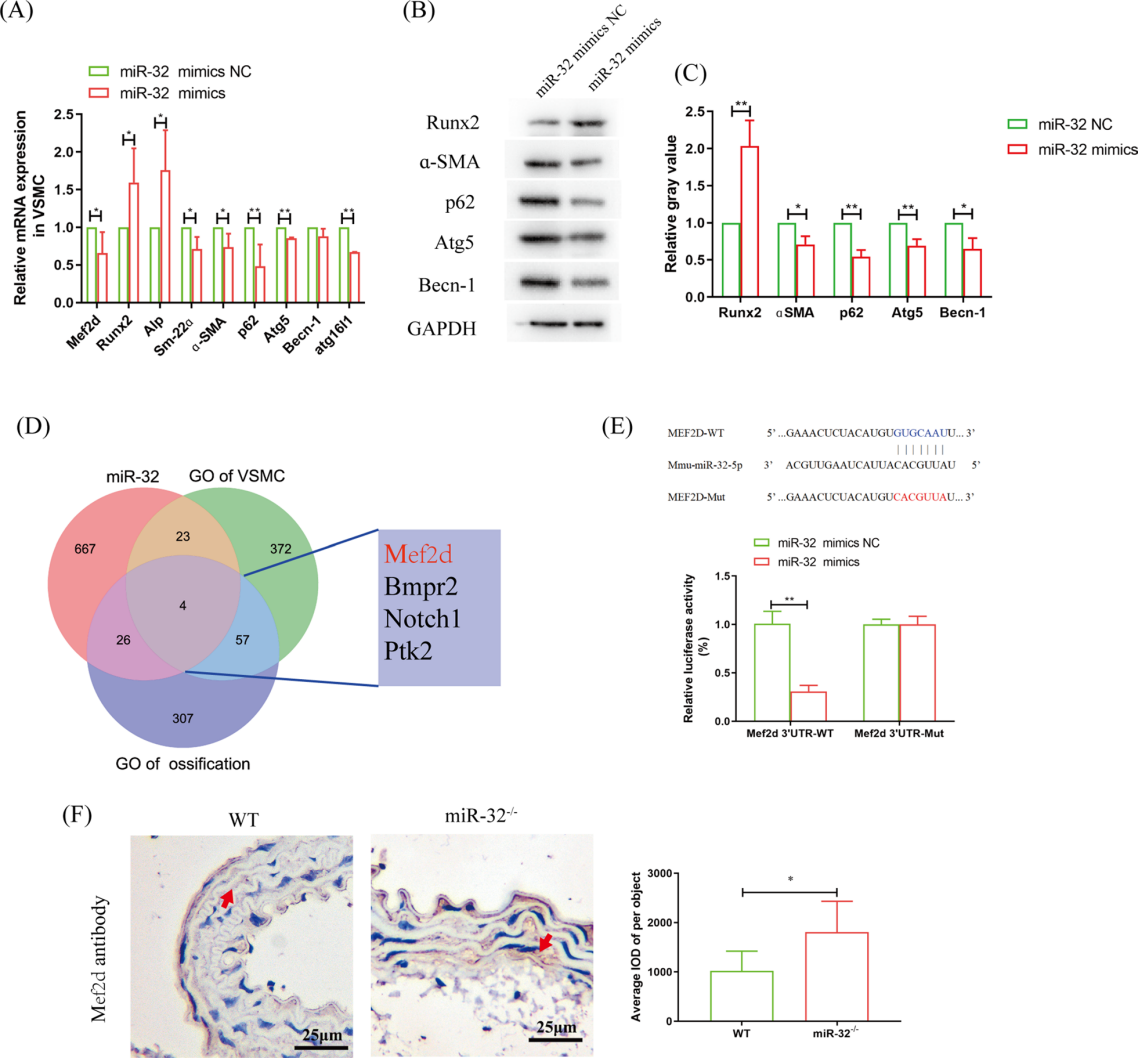
miR-32 promotes VSMC osteogenic differentiation maybe through targeting Mef2d. **A** QRT-PCR analyzed the autophagy and osteogenic differentiation of VSMC after miR-32 mimics transfection. **B**, **C** Western-blotting analyzed the autophagy and osteogenic differentiation oa VSMC after miR-32 mimics transfection (**B**), and statistical analysis (**C**). **D** Venn diagram analyzed the target gene of miR-32 via miRDB database and Gene Ontology database. **B** Luciferase assay analyzed the combination of miR-32 and Mef2d 3’-UTR. **F** Immunohistochemistry analyzed MEF2D expression in WT and miR-32^−/−^ mice. Aorta from 3 mice in the two groups were dissected and were used to immunohistochemical analysis, respectively. The cells experiment was repeated 3 times (*n* = 3), *p < 0.05 and **p < 0.01. Data was shown as means ± *s.e.m*

Our teams found that miR-32 is a multifunction molecular that is tightly related to cardiovascular diseases [7].. miR-32 was also increased in VSMCs after coculture with macrophages or treated with EVs secreted by macrophage under high glucose condition (Fig. 2B, C). After VSMCs were transfected with miR-32 mimics, the calcification marker genes (Runx2 and Alp) were significantly increased, and the marker genes of SMCs (Sm-22ɑ and ɑ-SMA) and autophagy (p62, Atg5, Becn-1 and Atg16l1) were all significantly decreased (Fig. 3A–C). These results suggest that EVs miR-32 plays an important role in the process of macrophage-promoted VSMC osteogenic differentiation.

### Mef2d antagonized the function of miR-32 in VSMCs

Furthermore, we analysed the target gene of miR-32 in VSMC osteogenic differentiation. Based on the miRDB database and Gene Ontology database, we found that Mef2d was the proper target gene (Fig. 3D), and a luciferase assay verified that miR-32 mimics transfection can significantly inhibited luciferase activation in the Mef2d 3’ UTR-WT group (Fig. 3E). In vitro, miR-32 mimics transfection significantly inhibited MEF2D expression in VSMCs (Fig. 3A); In vivo, we found that the expression of Mef2d was significantly increased in miR-32^−/−^ mice compared with WT mice (Fig. 3F).

**Fig. 4:**
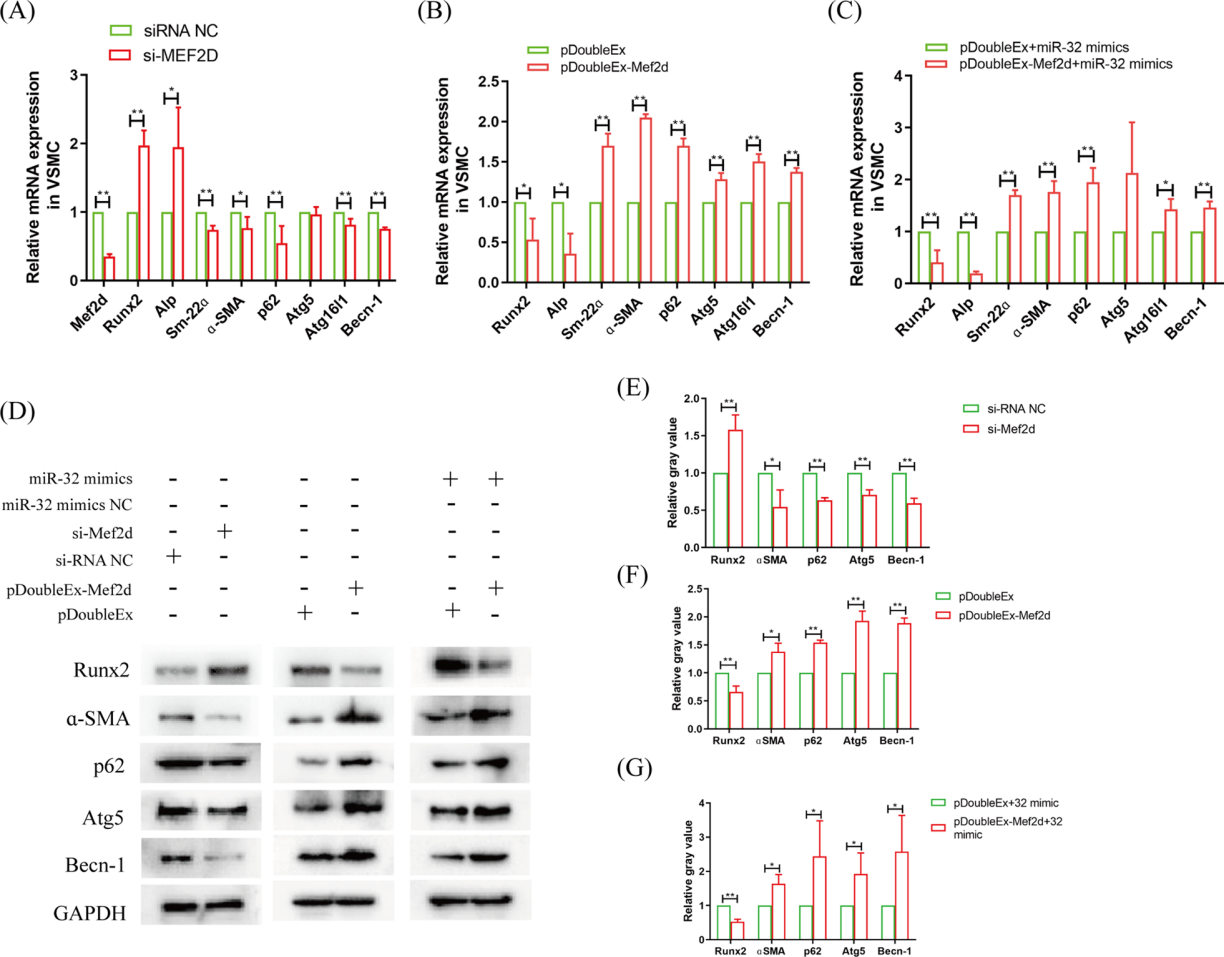
Mef2d antagonises the function of miR-32 in VSMC osteogenic differentiation. **A** qRT-PCR analyzed the autophagy and osteogenic differentiation of VSMC after si-Mef2d transfection. **B** qRT-PCR analyzed the autophagy and osteogenic differentiation of VSMC after pDoubleEx-Mef2d transfection. **C** qRT-PCR analyzed the antagonism function of Mef2d for miR-32 in VSMC after transfected with pDoubleEx-MEF2D and miR-32 mimics. **H** Western blotting analyzed the autophagy and osteogenic differentiation of VSMC after transfected with si-Mef2d or pDoubleEx-MEF2D or pDoubleEx-MEF2D and miR-32 mimics, and statistical analysis (**E**–**G**). The experiment was repeated 3 times (n = 3), *p < 0.05 and **p < 0.01. Data was shown as means ± *s.e.m*

Next, the roles of Mef2d were analysis in the process of VSMC autophagy and osteogenic differentiation were analysed. After VSMCs were transfected with si-Mef2d, the marker genes of calcification (Runx2 and Alp) were all significantly increased, and the marker genes of SMCs (Sm-22ɑ and ɑ-SMA) and autophagy (p62, Atg5, Becn-1 and Atg16l1) were all significantly decreased (Fig. 4A, D, E). When VSMCs overexpressed Mef2d, the marker genes of calcification were all significantly decreased, and the marker genes of SMC and autophagy were all significantly increased (Fig. 4B, D, F). Importantly, rescue experiments confirmed that Mef2d overexpression antagonized the function of miR-32 in VSMC autophagy and osteogenic differentiation (Fig. 4C, D, G). These results suggest that miR-32 regulates VSMC autophagy and osteogenic differentiation by targeting to Mef2d.

### cGMP/PKG is the key signalling pathway by which Mef2d regulates VSMC osteogenic differentiation

**Fig. 5:**
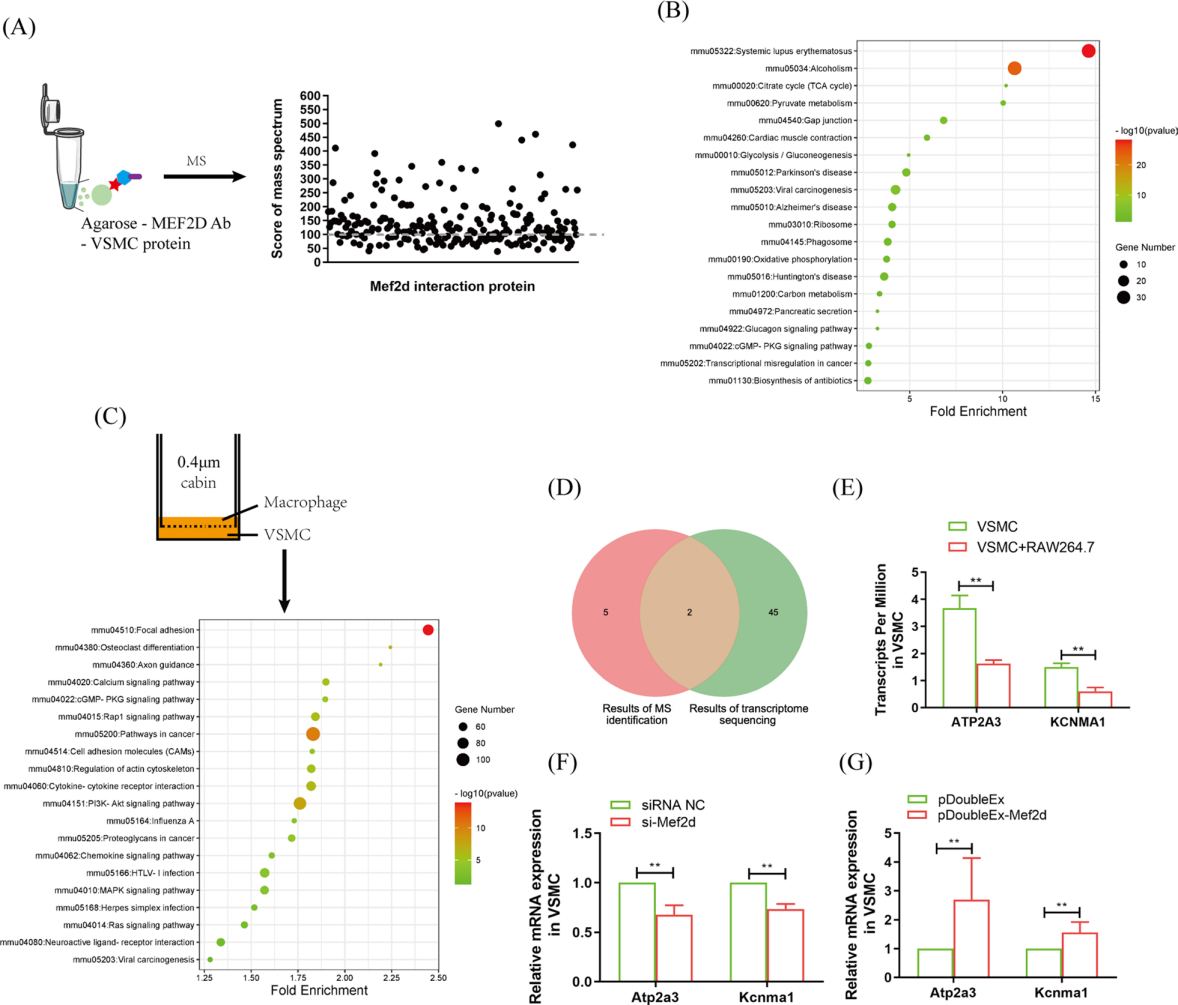
MEF2D regulates VSMC autophagy via cGMP-PKG signaling pathway. **A** Co-IP separated the interaction protein of MEF2D and identified by MS. **B** KEGG analyzed the signaling pathway of MEF2D interaction protein. **C** Transcriptome sequencing and KEGG analyzed the signaling pathway of macrophage regulated VSMC osteogenic differentiation. **D** Venn diagram analyzed the major protein of MEF2D regulated in cGMP-PKG signaling pathway. **E** The expression of Atp2a and Kcnma1 in VSMC after RAW264.7 treated. **F**, **G** The expression of Atp2a3 and Kcnma1 in VSMC after treated with si-Mef2d (**F**), or pDoubleEx-MEF2D (**G**). The experiment was repeated 3 times (n = 3), *p < 0.05 and **p < 0.01. Data was shown as means ± *s.e.m*

To reveal the mechanism by which Mef2d regulates autophagy, the proteins that interacted with Mef2d were separated using Co-IP and were identified using mass spectrometry (MS) (Fig. 5A, Additional file 1). A total of 159 proteins were identified. They were involved in 26 pathways according to DAVID cluster analysis, and 20 of them appeared to be enriched dot bubbles (Fig. 5B). In addition, VSMCs cocultured with macrophages were used for transcriptome sequencing analysis (Additional file 2). The DAVID cluster was used to analyse proteins that were significantly altered by more than twofold. In the top 20 pathways, the cGMP-PKG signalling pathway and viral carcinogenesis were the common signalling pathways regulated by Mef2d (Fig. 5B, C, Additional files 1, 2), and the cGMP-PKG signaling pathway was selected for further research due to its noticeable function in T2D-related cardiovascular diseases [27]. Then, Venn diagram was used to explore the common genes in the cGMP-PKG signalling pathway. The results reveled two common genes, ATPase sarcoplasmic/endoplasmic reticulum Ca^2+^ transporting 3 (Atp2a3) and potassium calcium-activated channel subfamily M alpha 1 (Kcnma1) (Fig. 5D). The expression trend of these two genes was consistent with that of Mef2d in VSMCs after coculture with macrophages (Fig. 5E). Moreover, pDoubleEx-MEF2D transfection promoted Atp2a3 and Kcnma1 expression in VSMCs, while si-Mef2d transfection inhibited the expression of these genes (Fig. 5F, G). These findings suggest that the cGMP-PKG signalling pathway is crucial for Mef2d regulation of VSMC autophagy.

### Macrophage EVs miR-32 promoted aortic calcification by inhibiting autophagy in miR-32.^−/−^ T2D mice

**Fig. 6:**
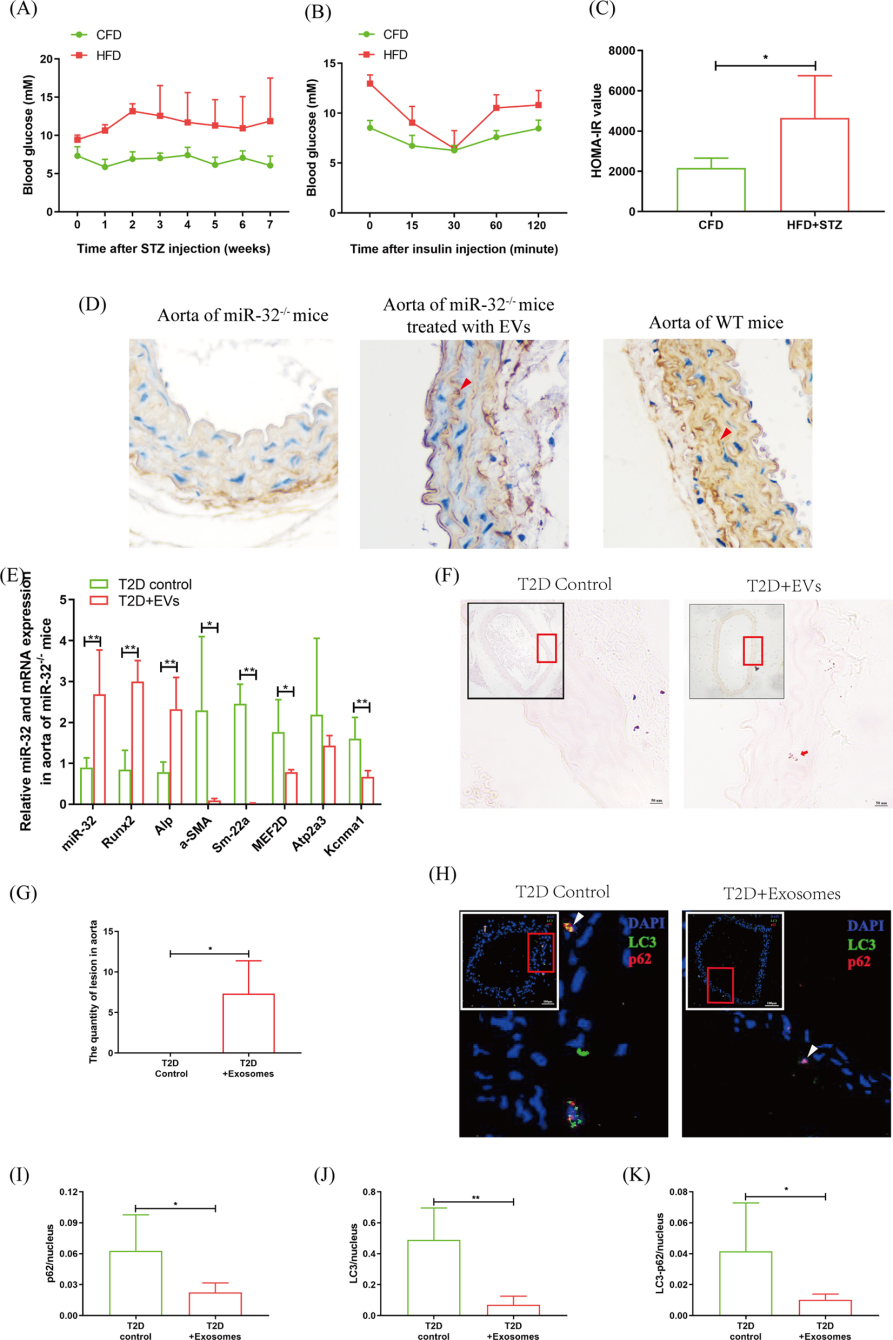
In vivo analysis of macrophage EVs miR-32 promotes aorta calcification by inhibited autophagy. Analysis of VSMC autophagy and osteogenic differentiation in the aorta of miR-32^−/−^ T2D mice after treated with macrophage EVs. **A** Blood glucose analysis. **B** IPITT analysis. **C** HOMA-IR analysis. **D** Immunohistochemisty analyzed miR-32 expression in aorta of miR-32^−/−^ mice, and WT mice, and miR-32^−/−^ injection EVs. **C** qRT-PCR analyzed aorta osteogenic differentiation. **F**, **G** alizarin red staining analyzed aorta calcification after macrophage EVs injection (**F**), and statistical analysis (**G**). **H–K** Immunofluorescence analyzed p62 and LC3 expressions in aorta (**H**), and statistical analyzed the puncta of p62 (**I**), LC3 (**J**), and p62-LC3 (**K**). 3 per 5 mice in different groups were used in the detection. *p < 0.05 and **p < 0.01. Data was shown as means ± *s.e.m*

In vivo, miR-32^−/−^ mice were used to establish a T2D mouse model. Compared with CFD mice, the model mice had marked characteristics of T2D, such as highblood glucose levels and insulin resistance (Fig. 6A–C). Then, the T2D mice were injected with EVs secreted by macrophages under high glucose conditions, and miR-32 appeared in the VSMCs of the aorta (Fig. 6D). Moreover, in EVs-treated miR-32^−/−^ T2D mice, the expression of miR-32 and the ossification genes (Runx2 and Alp) were all significantly increased, and the marker genes of SMCs (ɑ-SMA and Sm-22ɑ) and the cGMP-PKG signalling pathway (Atp2a3 and Kcnma1) were all significantly decreased (Fig. 6E). In addition, autophagy analysis revealed that p62, LC3, and p62-LC3 puncta were all significantly decreased in the aortas of miR-32^−/−^ mice after treatment with EVs (Fig. 6F–I). These findings suggest that miR-32 is an important molecule in macrophage EVs that promotes aortic calcification in T2D mice.

## Discussion

Diabetes is a chronic disease that is characteried by impaired glucose metabolism. This condition can easily cause microvascular and macrovascular complications due to high glucoselevels that can easily active the immune system [28, 29], especially the activation of macrophages [30]. Based on our previous reports on miR-32 in coronary artery calcification and microglia [7, 8], this study further explored the roles and mechanisms by which miR-32 acts in the process of macrophage-regulated T2D VC.

High glucose is one of the major pathological factors associated with diabetes [31], and it easily leads to the dysregulation of the functions of inflammatory and immune cells, including macrophages [16]. Under high glucose conditions, we found that macrophages preferentially polarized to the M1 phenotype (Fig. 1A, B). M1 macrophages constitute the primary immune cell-mediated inflammatory reaction, which is one of the leading causes of VC [32]. However, in addition to producing inflammatory factors, secreting EVs is another crucial pathway by which macrophages drive the function of immune cells [33, 34]. Furthermore, EVs are key carriers of miRNAs [35]. Interestingly, we found that high glucose conditions promoted miR-32 expression not only in macrophages but also in their EVs (Fig. 1C). Furthermore, macrophages and EVs all significantly increased miR-32 expression in VSMCs and their osteogenic differentiation (Fig. 2). Therefore, these findings suggest that macrophage EVs promote VSMC osteogenic differentiation and that miR-32 may drive the function of EVs under high-glucose conditions.

Next, we identified that Mef2d, which is involved in the autophagy process [36–38], is the key target gene of miR-32 in VSMC osteogenic differentiation (Figs. 3, 4). To illustrate the regulatory pathway of Mef2d in VSMC autophagy, we identified the protein that interacts with Mef2d and then analysed the change in transcription levels in VSMCs cocultured with with macrophage under high glucose conditions. Cluster analysis showed that the cGMP-PKG was the common signalling pathway, and Ato2a3 and Kcnma1 were the common proteins in cGMP-PKG (Fig. 5A–E). Impaired cGMP-PKG signalling causes cardiomyocyte stiffening in diabetic hearts. and the symptoms can be modulated through restoration of the normal kinase activities of PKG by neuregulin-1 [27]. In particular, cGMP-PKG is tightly correlated with diabetes vascular dysfunction and cardiomyocyte autophagy [39–41]. A reduction in autophagy leads to cardiac dysfunction, including cardiovascular calcification [42, 43]. This evidence highlights the important relationship between the cGMP-PKG signalling pathway and VSMC autophagy. Further research found that the expression of Atp2a3 and Kcnma1 was consistent with the expression change in Mef2d expression (Fig. 5F, G). Knockout of Kcnma1 leads to autophagic dysfunction [44]. Therefore, these findings suggest that Mef2d mediates VSMC autophagy by regulating the cGMP-PKG signalling pathway.

EVs transplantation is typically used to analyse the function of miRNAs in cell–cell crosstalk [45]. After T2D miR-32^−/−^ mice were injected with miR-32 EVs, miR-32 appeared in the aortic (Fig. 6D), and aortic calcification was significantly increased (Fig. 6E–G). One important cause of VSMC calcification is impaired autophagy [24], and the changes in LC3, p62, and LC3-p62 dots represent autophagosome formation [46]. In this study, EVs transplantation significantly promoted aortic calcification and inhibited autophagosome formation in the aortas of T2D miR-32^−/−^ mice (Fig. 6H–K). Thus, these findings suggest that macrophage EVs promote the development of T2D VC that miR-32 is a key regulator of this process.

Although miR-32 has been identified as an important molecule in macrophage EVs that promotes T2D VC, we cannot exclude the influence of other EVs compositions on T2D VC. Currently, some reports attempted to resolve this problem. Luo ZL et al. eliminated DNA in EVs through electroporation and DNase I treatment [47]. Therefore, the feasibility of electroporation and miRNA inhibitor treatment will promote research on EVs function.

## Conclusions

Our findings demonstrated that high glucose conditions promote macrophage M1 polarization by upregulating miR-32 and increasing miR-32 levels in EVs. Furthermore, EVs miR-32 promotes aortic calcification in T2D miR-32^−/−^ mice through miR-32/Mef2d/cGMP-PKG-mediated VSMC autophagy. These findings illustrate an important mechanism by which macrophages promote VC in T2D and identify a candidate biomarker of T2D VC.

**Table 1:** Primers sequence

Gene name	Primer sequence (5’→3’)
miR-32	CGCGCTATTGCACATTACTAAGTTGCA
Mef2d-F	GGCTGGCACTAGGCAATGTCAC
Mef2d-R	CTGCTGTGGCTGTGGCTGTG
iNOS-F	ACTCAGCCAAGCCCTCACCTAC
iNOS-R	TCCAATCTCTGCCTATCCGTCTCG
TNFɑ-F	GCGACGTGGAACTGGCAGAAG
TNFɑ-R	GCCACAAGCAGGAATGAGAAGAGG
p62-F	AGGAGGAGACGATGACTGGACAC
p62-R	TTGGTCTGTAGGAGCCTGGTGAG
Atg5-F	TGCGGTTGAGGCTCACTTTATGTC
Atg5-R	GTCCCATCCAGAGCTGCTTGTG
Becn-1-F	AGGCAGTGGCGGCTCCTATTC
Becn-1-R	TGAGGACACCCAGGCAAGACC
Atg16l1-F	CAAGCCGAATCTGGACTGTGGATG
Atg16l1-R	CGGTCGTGACTTCCTGAGACAATC
Runx2-F	AGTCCCAACTTCCTGTGCT
Runx2-R	CTGCTCCGTTCTCAAAGTGG
Bglap1-F	CAAGCAGGAGGGCAATAAGGTAGTG
Bglap1-R	CATACTGGTCTGATAGCTCGTCACAAG
Alp-F	CACGGCGTCCATGAGCAGAAC
Alp-R	CAGGCACAGTGGTCAAGGTTGG
SM22ɑ-F	GCCTGAGAACCCACCCT
SM22ɑ-R	CGAAACCCGTCAAACCG
αSMA-F	GGCATCCACGAAACCACCTAT
αSMA-R	ATGAGACAGACCTAGCCACCG
GAPDH-F	TGTTTCCTCGTCCCGTAG
GAPDH-R	CAATCTCCACTTTGCCACT

**Table 2:** Antibodies used in the experiments

Antibody name	Source	Manufacturer	Catalog # (RRID)	Application
Anti-F4/80-APC	Rat	Thermo Fisher Scientific	17-4801-80#AB_2784647	IF(1:50)
Anti-CD11b-Percp-Cy5.5	Rat	Thermo Fisher Scientific	45-0112-80#AB_953560	IF(1:50)
Anti-CD86-FITC	Rat	Thermo Fisher Scientific	11-0862-82#AB_465148	IF(1:50)
Anti-iNOS	Rabbit	proteintech	18985-1-AP#AB_2782960	IHC(1:100)
Anti-F4/80	Rat	Thermo Fisher Scientific	14-4801-82#AB_467558	IHC(1:100)
ɑ-SMA	Rabbit	Cell signaling	19245#AB_2734735	WB(1:1000)
Runx2	Rabbit	abcam	20700-1-AP#AB_2722783	WB(1:500)
Anti-CD63	Rabbit	Thermo Fisher Scientific	PA5-92370#AB_2806456	WB(1:1000)
Anti-TSG101	Rabbit	Thermo Fisher Scientific	PA5-82236#AB_2789397	WB(1:1000)
Anti-Mef2d	Rabbit	Proteintech	14353-1-AP#AB_2878046	WB(1:1000) IHC(1:100)
Anti-p62	Rabbit	Proteintech	18420-1-AP#AB_10694431	WB(1:1000)
Anti-p62	mouse	Proteintech	66184-1-Ig#AB_2881579	IFC(1:100)
Anti-Atg5	Rabbit	Proteintech	10181-2-AP#AB_2062045	WB(1:1000)
Anti-Becn-1	Rabbit	Proteintech	11306-1-AP#AB_2259061	WB(1:1000)
Anti-LC3	Rabbit	Proteintech	14600-1-AP#AB_2137737	IFC(1:100)

## Supplementary Information

**Additional file 1**. MS analyze the interaction protein of Mef2d.

**Additional file 2**. Transcriptome sequencing analyze the influence of macrophage for genes expression in VSMC.

## Data Availability

All data in the article can be requested from the corresponding author.
